# Depth-related variability in viral communities in highly stratified sulfidic mine tailings

**DOI:** 10.1186/s40168-020-00848-3

**Published:** 2020-06-09

**Authors:** Shao-Ming Gao, Axel Schippers, Nan Chen, Yang Yuan, Miao-Miao Zhang, Qi Li, Bin Liao, Wen-Sheng Shu, Li-Nan Huang

**Affiliations:** 1 School of Life Sciences, Sun Yat-sen University, Guangzhou, 510275 People’s Republic of China; 2 Resource Geochemistry, Federal Institute for Geosciences and Natural Resources (BGR), Stilleweg 2, 30655 Hannover, Germany; 3 School of Life Sciences, South China Normal University, Guangzhou, 510631 People’s Republic of China

**Keywords:** Stratified mine tailings, Viruses, Diversity, Functions, Auxiliary metabolic genes

## Abstract

**Background:**

Recent studies have significantly expanded our knowledge of viral diversity and functions in the environment. Exploring the ecological relationships between viruses, hosts, and the environment is a crucial first step towards a deeper understanding of the complex and dynamic interplays among them.

**Results:**

Here, we obtained extensive 16S rRNA gene amplicon, metagenomics sequencing, and geochemical datasets from different depths of two highly stratified sulfidic mine tailings cores with steep geochemical gradients especially pH, and explored how variations in viral community composition and functions were coupled to the co-existing prokaryotic assemblages and the varying environmental conditions. Our data showed that many viruses in the mine tailings represented novel genera, based on gene-sharing networks. *Siphoviridae*, *Podoviridae*, and *Myoviridae* dominated the classified viruses in the surface tailings and deeper layers. Both viral richness and normalized coverage increased with depth in the tailings cores and were significantly correlated with geochemical properties, for example, pH. Viral richness was also coupled to prokaryotic richness (Pearson’s *r* = 0.65, *P* = 0.032). The enrichment of prophages in the surface mine tailings suggested a preference of lysogenic viral lifestyle in more acidic conditions. Community-wide comparative analyses clearly showed that viruses in the surface tailings encoded genes mostly with unknown functions while viruses in the deeper layers contained genes mainly annotated as conventional functions related to metabolism and structure. Notably, significantly abundant assimilatory sulfate reduction genes were identified from the deeper tailings layers and they were widespread in viruses predicted to infect diverse bacterial phyla.

**Conclusions:**

Overall, our results revealed a depth-related distribution of viral populations in the extreme and heterogeneous tailings system. The viruses may interact with diverse hosts and dynamic environmental conditions and likely play a role in the functioning of microbial community and modulate sulfur cycles in situ.

Video Abstract

## Background

Viruses are abundant and critical components of microbial communities in the environment [1]. Historically, studies of viral diversity have largely relied on culture-dependent techniques with well-recognized limitations, including especially the inconsistency between morphological and genetic taxon identification [2]. While marker gene surveys have revolutionized our understanding of cellular systematics and diversity, such approaches cannot be adopted in viral ecology studies due to the absence of a phylogenetically informative universal marker owing to the mosaic nature of viral genome organization [3]. To tackle these problems, recent works have employed metagenomic sequencing to discover viral sequences from a wide variety of habitats including marine and freshwater environments [3–5], soils [6, 7], and extreme environments [8–10]. These studies often reveal the existence of diverse viral assemblages in nature, whose members remain largely uncharacterized (unknown virosphere), and significantly improve our understanding of the ecological roles of viruses in Earth’s major ecosystems [4, 11]. A current challenge is to move beyond the two basic questions, i.e., what is there and what is it doing, to a more in-depth analysis of the dynamic interplay between viruses, microbes, and environmental conditions [12].

Viruses can substantially affect the ecology, evolution, and physiology of their hosts in natural settings by causing host mortality, facilitating horizontal gene transfer, and influencing biogeochemical cycles via production of dissolved organic matter through cell lysis or participating in host metabolisms with auxiliary metabolic genes (AMGs) [13, 14]. In the meantime, viruses are intracellular obligatory parasites that repurpose the host cell machinery to replicate; thus, prokaryotic hosts play a key role in regulating viral populations [9]. Population oscillations of viruses and their hosts have been documented [5] and reviewed [15] in natural and cultivated environments. Furthermore, geochemical conditions may also have a significant influence on viral populations via direct or indirect mechanisms. Analyses of viruses in the pelagic upper-ocean revealed that viral communities are locally structured by environmental conditions that affect host community structure [16]. Additionally, the AMGs in viral genomes are obtained by horizontal gene transfer from their hosts and exhibit parallel depth-stratified host adaptations [14]. All these aspects imply a complicated interaction between viruses, hosts, and environments.

Acid mine drainage (AMD) is a worldwide environmental problem that arises largely from microbially mediated oxidative dissolution of sulfidic ores exposed to oxygen and water during mining activities [17]. These environments are characterized by low pH and high concentrations of metals and sulfate, representing an extreme environment for life. AMD environments are well recognized as model systems for the study of microbial community structure, functions, and evolution due to their reduced complexity and have been studied extensively by cultivation-independent molecular approaches [18–20]. Meanwhile, several investigations with a specific focus on viruses in AMD systems have been reported. These early works documented a major influence of minerals (via attachment) on viral abundance [21, 22], unveiled the coevolution relationships between viruses and their specific hosts [23], and uncovered viruses infecting cells of the archaeal lineages of ARMAN and *Thermoplasmatales* [24]. In contrast, while waste tailings dumps are an important source of AMD around the globe [20], relatively little is known about the microbial diversity and ecology in these harsh, highly heterogeneous environments [25, 26], and the indigenous viral communities have never been investigated. Mine tailings dumps are typically stratified into distinct geochemical zones, reflecting progressive oxidation of sulfide minerals in the tailings and indicating that each of these zones is shaped by organisms with specific metabolic traits [26]. Thus, mine tailings offer unique possibilities to resolve complex biological interactions and to explore the relationship between these dynamic interactions and multivariate geochemistry.

Here, we report the analysis of two highly stratified tailings cores sampled from a sulfidic tailings impoundment of a Pb/Zn mine where extremely low pH and metal-rich drainage is a persistent feature. The composition of both the prokaryotic and viral populations in different sections of the cores was resolved by 16S rRNA gene high-throughput sequencing and recovering viral sequences from metagenomic datasets, respectively. We assessed how prokaryotic and viral communities varied along the tailings depth profiles and examined how the down-core stratification of viral diversity and functions were related to the co-existing prokaryotic assemblages and tailings geochemistry.

## Results

### Physicochemical stratification of mine tailings

**Fig. 1:**
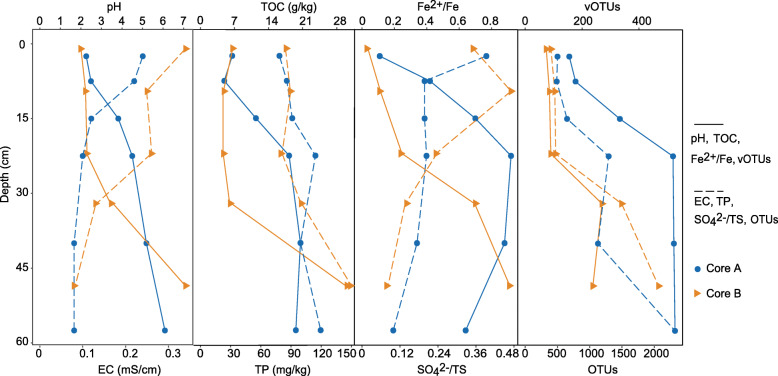
Vertical profiles of physicochemical and biodiversity data for the two tailings cores from the Fankou Pb/Zn mine located in Guangdong Province, P.R. China. The intermediate depth of each layer is taken as the depth of each sample. EC, electronic conductivity; TOC, total organic carbon; TP, total phosphorus; TS, total sulfur; vOTUs, the number of viral operational taxonomic units; OTUs, the number of prokaryotic operational taxonomic units

Both tailings cores showed steep vertical gradients of physicochemical properties (Fig. 1). pH values shifted from extremely acidic at the surface layers to near neutral at the deeper layers, while electronic conductivity (EC) declined with depth along the vertical profiles. Both total organic carbon (TOC) and total phosphorus (TP) exhibited an increase with depth. The ratio of Fe^2+^ to total Fe increased dramatically with depth, contrasting to the decrease in the ratio of SO_4_^2−^ to total sulfur (TS). This indicated a shift from an oxidative environment at the surface tailings to a reductive condition at the deeper layers. Additionally, differences in geochemistry between the two mine tailings cores were also evidenced. For detailed physicochemical parameters of the tailings samples, see Additional file 1: Table S1 in the Supplemental material.

### Diversity and distribution of viral and prokaryotic communities

**Fig. 2:**
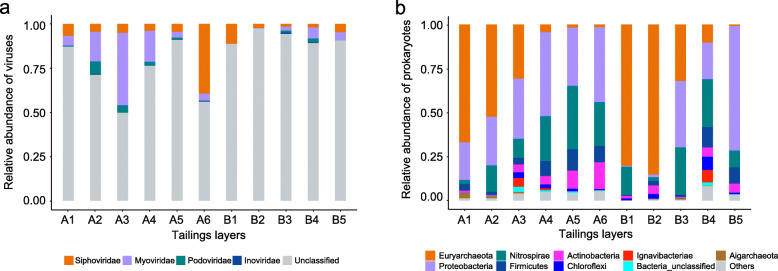
Relative abundance of **a** viruses (family level) and **b** prokaryotes (phylum level) in the 11 depth-stratified mine tailings layers revealed by metagenomics and 16S rRNA gene amplicon sequencing, respectively

Application of viral protein family-based pipeline [27] and VirSorter software [28] to predict viral sequences in the two cross-assembled metagenomic assemblies led to the identification of 844 putative DNA metagenomic viral scaffolds. Of these, 631 were identified by only one tool and 213 identified by both tools (Additional file 1: Table S2). All viral scaffolds were grouped at approximately the species level into 750 viral operational taxonomic units (vOTUs), of which 116 could be taxonomically affiliated and corresponded to double-stranded DNA (dsDNA) and single-stranded DNA (ssDNA) viruses (Additional file 1: Table S3). The number of vOTUs in each tailings layer ranged from 74 to 527 and generally increased with depth (Fig. 1 and Additional file 1: Table S1). Examination of relative abundance of viruses in each tailings layer (calculated as the cumulative normalized coverage of its members divided by the total normalized coverage of viruses in that community) showed that the classified viruses accounted for 2.56~50.2% of all viral communities, most of which were assigned to one of the three families (*Myoviridae*, *Siphoviridae*, and *Podoviridae*) in the *Caudovirales* order (Fig. 2a and Additional file 1: Table S3).

The barcoded 16S rRNA gene sequencing generated 1,742,197 quality sequences from the 11 tailings samples, with a range of 43,424 to 134,565 sequences per community (Additional file 1: Table S4). A total of 3371 phylotypes were defined at a 97% sequence similarity cutoff; most (99%) of which could be assigned to a taxonomic group (phylum) by the RDP classifier (80% threshold). The prokaryotic phylotype richness generally increased with depth (ranging from 398 to 2321 in each sample), coincident with the vertical distribution of viral diversity. Examination of the relative abundance of the dominant lineages showed contrasting patterns: while archaeal phylotypes were most abundant in the surface tailings layers, those of bacteria were most frequently detected in the deeper layers. Specifically, *Euryarchaeota* represented 67% and 80% of the total sequences of the surface tailings (A1 and B1, respectively), whereas *Proteobacteria*, *Nitrospirae*, and *Firmicutes* collectively accounted for 77% and 90% of the total communities in the deeper layers (A6 and B5, respectively) (Fig. 2b).

### Correlations between viral communities, prokaryotic communities, and geochemical data

**Fig. 3:**
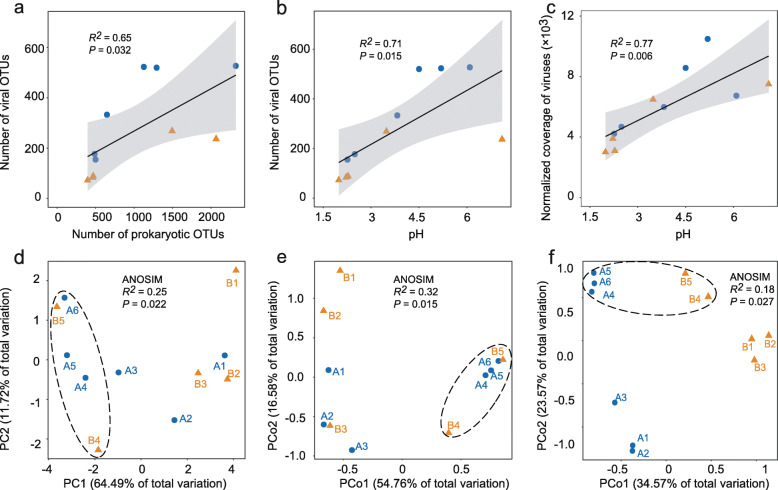
**a**–**c** Significant Pearson correlations between prokaryotic community, viral community, and pH. **d** Principal component analysis (PCA) of geochemical data as derived from Euclidean dissimilarities, and principle coordinate analysis (PCoA) of **e** prokaryotic communities and **f** viral communities as derived from Bray–Curtis dissimilarities. The analysis of similarity (ANOSIM) statistics considers samples grouped by depth (inside and outside the dashed ellipses)

Significant correlations were observed between viral community composition, prokaryotic community composition, and geochemical data (Additional file 2: Fig. S1). Specifically, the two tailings cores exhibited similar increases in the number of vOTUs with increasing prokaryotic richness along the depth profiles as expected (Fig. 1 and Fig. 3a). Meanwhile, the number of vOTUs and the overall normalized coverage of viruses were also significantly correlated with measured geochemical parameters, for example, pH (Fig. 3b, c).

Euclidean distance-based principal component analysis (PCA) and Bray–Curtis distance-based principle coordinate analysis (PCoA) were applied to further reveal the clustering patterns of physicochemical properties (Fig. 3d), and prokaryotic communities (Fig. 3e), and viral communities (Fig. 3f) of the tailings, respectively. Results showed that physicochemical properties and prokaryotic and viral communities (OTU level) of samples from the vertical profiles of the tailings cores were apparently separated between surface and deeper layers, indicating a significant depth-related variability in the biotic and abiotic signals and the potential correlations between them. In support of this, Mantel test analysis revealed that viral community dissimilarity (estimated between all pairwise combinations of samples) increased with an increasing difference in the prokaryotic community (Mantel’s *r* = 0.47, *P* = 0.004) and geochemical characteristics (Mantel’s *r* = 0.43, *P* = 0.005). Meanwhile, both prokaryotic and viral community dissimilarities were most significantly related to EC (Additional file 2: Fig. S2). Notably, viral communities were also apparently separated between the two tailings cores (ANOSIM *R*^*2*^ = 0.24, *P* < 0.01) (Fig. 3f), mirroring the between-core differences in geochemical properties (Fig. 1).

**Fig. 4:**
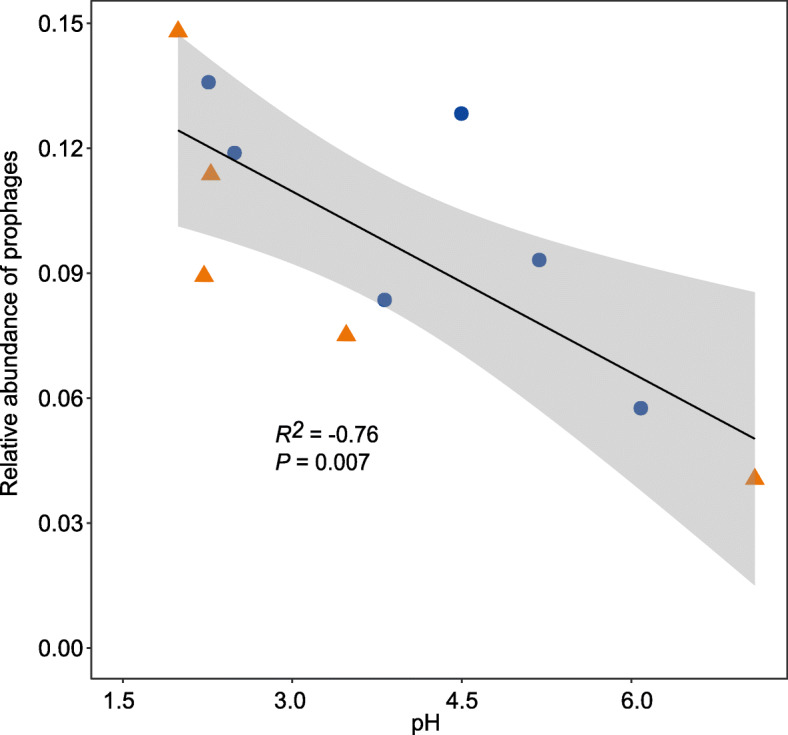
Significant Pearson correlations between pH and the relative abundance of prophages in the 11 mine tailings layers

Next, we performed extensive genome reconstruction for the bacteria and archaea present in the tailings cores to resolve putative hosts of the identified viruses. This resulted in a total of 435 draft prokaryotic genomes. These genomes were then screened for genomic features linking viruses to potential hosts. Protospacers were identified in 4 predicted viral scaffolds, and 22 prophages were matched to their putative hosts (Additional file 1: Table S5). Together, putative hosts from 11 bacterial and archaeal phyla were predicted for 26 viral scaffolds (Additional file 1: Table S5). Notably, the total relative abundance of prophages exhibited a depth-related distribution in the two cores and correlated with pH significantly (Pearson’s *r* = − 0.76, *P* = 0.007) (Fig. 4).

### Community-wide comparative gene profiles

**Fig. 5:**
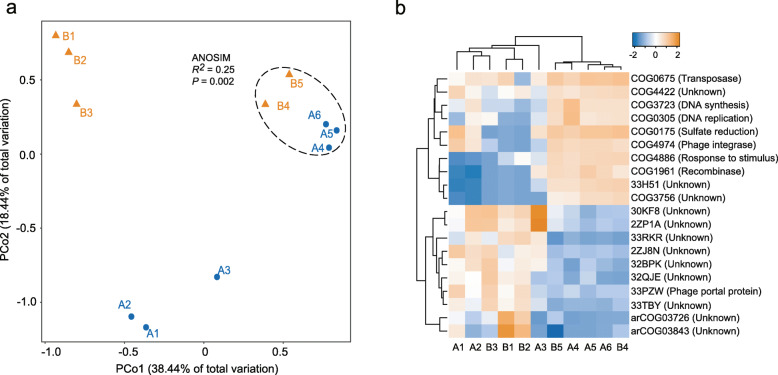
Overview of functional profiles of viral communities. **a** PCoA of viral COGs based on their relative abundance in each community. The analysis of similarity (ANOSIM) statistics considers samples grouped by depth (inside and outside the dashed ellipse). **b** Hierarchical clustering of the top ten abundant indicator COGs in the surface and the deeper layers, respectively. Relative abundances were log transformed and normalized with a *z*-score method

To explore the metabolic capabilities and functional diversity of viral communities associated with different depths, cluster of orthologous group (COG) annotation of viral genomes was performed by comparing the predicted viral proteins against the eggNOG database (5.0.0) (Additional file 1: Table S6) [29], and the normalized coverage of each COG was calculated. Bray–Curtis distance-based PCoA again revealed strong primary clustering of viral COGs by depth (Fig. 5a). Further analysis indicated that 60 out of 2002 COGs displayed significantly (*P* < 0.05) different normalized coverage between the surface tailings and deeper layers (Additional file 1: Table S7). We defined a COG with a significantly higher or lower normalized coverage in the surface tailings than that in the deeper layer viral communities as an indicator COG. Accordingly, 20 indicator COGs on 49 viral sequences and 40 indicator COGs on 426 viral sequences were identified for the surface communities and deeper layer communities, respectively (Additional file 1: Table S7). Meanwhile, virus-specific genes (defined in the “Methods” section) and short genes (< 1 kb) were found on most of these viral sequences (Additional file 1: Table S8). Interestingly, most of the indicator COGs in the surface tailings could not be assigned to any known functions (Fig. 5b and Additional file 1: Table S7). Additionally, two indicator COGs were assigned as virus-specific functions that are mainly involved in the synthesis of phage portal protein (33PZW) and archaeal phage integrase (arCOG01244) (Fig. 5b and Additional file 1: Table S7). In contrast, the deeper layer viral communities harbored a large proportion of higher indicator COGs related to assimilatory sulfate reduction (ASR) (COG0175), transposase (COG0675), DNA replication (initiation and elongation, COG0305), DNA synthesis (COG3723), phage integrase (COG4974), and recombinase (COG1961) (Fig. 5b and Additional file 1: Table S7). To further examine potential links between viral functions and community structure, we analyzed the relative abundance and composition of viral genomes that encoded the indicator COGs in each tailings layer. These viruses accounted for a significant proportion of the total viral communities in the surface and deeper layers (37.6% and 72.1% in the B2 and A6 communities, respectively) (Additional file 2: Fig. S3). Taxonomic classification of these viral genomes further revealed that families of *Caudovirales* order, which constitute largely the classifiable viruses in the mine tailings, primarily encoded the indicator COGs in all layers (Additional file 1: Table S8).

### Case study of AMGs

**Fig. 6:**
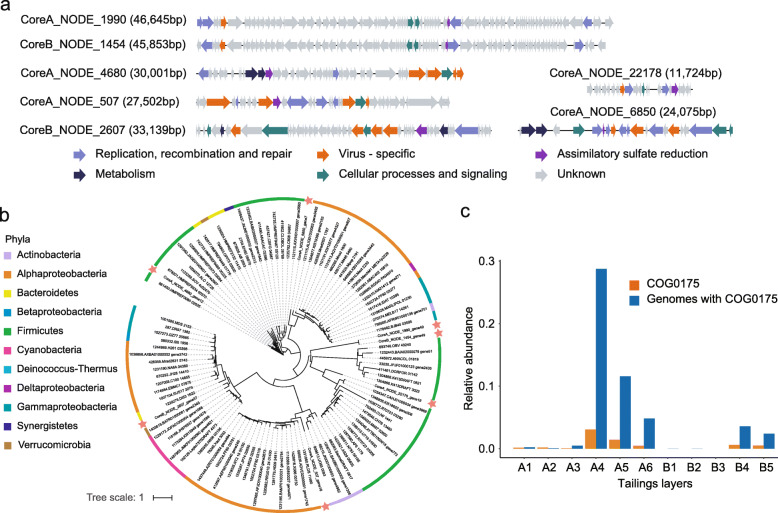
Genomic analysis of viral sulfate reduction genes. **a** Genome map of seven viral scaffolds containing assimilatory sulfate reduction genes. Genes related to replication, recombination, and repair are shown in light purple; genes related to metabolism are shown in dark blue; genes related to cellular processes and signaling are shown in green; viral hallmark genes are in orange; and assimilatory sulfur reduction genes (COG0175) are in dark purple and unknown genes are in grey. Detailed function descriptions of the seven viral scaffolds are listed in Additional file 1: Table S9. **b** Maximum-likelihood phylogenetic tree with assimilatory sulfate reduction genes from mine tailings viral genomes (indicated by stars) compared to genes found in bacterial reference sequences (the “Methods” section). The scale bar represents 1 amino acid substitution per site. **c** Total relative abundance of COG0175 and genomes containing COG0175 in each tailings layer viral genome

Having illustrated the community-wide functional profiles, we next sought to identify the putative virus-encoded AMGs that could modify host metabolism during infection. Given the observed lower ratio of SO_4_^2−^/TS (Fig. 1) and higher abundance of ASR (COG0175) genes in the deeper tailings layers (Fig. 4b), genes related to ASR were selected for subsequent analysis. We found seven predicted viral scaffolds that harbored genes participating in ASR (COG0175) (Fig. 6a and Additional file 1: Table S9), which are important for the reduction of 3′-phosphoadenosine 5′-phosphosulfate (PAPS) and the conversion of sulfate to sulfite [30]. All these predicted viral scaffolds were longer than 10 kb and contained one or more virus-specific genes (Fig. 6a). One of them (CoreA_NODE_507) was predicted as prophage with its ASR gene flanked by viral-specific genes (Fig. 6a).

To further explore the origin of these predicted viral genes, 99 homologs from 11 prokaryotic phyla were recruited and combined to build a phylogenentic tree (Fig. 6b and Additional file 1: Table S10), and their putative hosts were predicted as nearest neighbors. The phylogenetic analysis showed that the sulfate reduction genes in the viral genomes “CoreA_NODE_22178” and “CoreA_NODE_4680” clustered with their counterparts from *Firmicutes*, indicating that these AMGs might be acquired from this widely distributed bacterial lineage. This result was in agreement with our prediction of *Firmicutes* as the putative host of the viral genome “CoreA_NODE_22178” (Additional file 1: Table S5). However, the hosts of other ASR genes were uncertain, as they clustered with homologous genes from different phyla (Fig. 6b). Nonetheless, reads mapping to the seven predicted viral genomes and their predicted ASR genes showed that both these genomes and genes were significantly (Wilcoxon *t* test, *P* < 0.01) enriched in the deeper layers of the tailings cores (Fig. 6c), implying a potential impact of viral ASR on the sulfur cycles in situ.

## Discussion

The highly stratified physicochemical and biological profile in the Fankou Pb/Zn sulfidic mine tailings has enabled an in-depth exploration of the variation of viral communities in the context of geochemical changes. While many viral ecology studies have employed size-based enrichment of viral particles from environmental (especially aqueous) samples to generate the metagenomes (viral metagenomes or viromes) [3–5], such strategies are particularly challenging when it comes to soil and sediments due to the difficulties in isolating soil viruses and the distinct properties between sites that prevent generalization [31]. Thus, we performed metagenomic sequencing on total genomic DNA extracted directly from the mine tailings, which would allow recovery of sequences from not only temperate viruses that are either integrated into host genomes or present as episomal elements in the host cells, but also free virus particles present in the original samples [32]. Consequently, without enrichment of free viruses adsorbed to soil and breaking open viral capsids, the predicted viral sequences in our study are likely biased towards intracellular viruses [31, 32]. While metagenomics has brought new opportunities to the rapidly progressing field of viral ecology, identification of putative viral sequences in the sequence datasets remains a major challenge. Previous studies have employed viral protein families and VirSorter software [1, 33]. However, benchmarking of the two computational approaches demonstrated that the viral protein family-based pipeline had a better precision whereas the recall rate was higher with VirSorter in a synthetic metagenome [1]. These rarely behave in a similar way to metagenomes from natural communities. Thus, we employed separately these methods in our study and merged the identified viral scaffolds data, uncovering a large proportion of unclassified viral genomes in the Fankou mine tailings (Fig. 2a). Unknown virospheres have recently been discovered in many other habitats such as marine environments, acidic hot springs, and permafrost soils [4, 8, 9]. Given that the reticulate classification method of viral sequences uses shared gene content information [2, 34] and that currently the isolated archaeal viruses are largely outnumbered by bacteriophages [35, 36], it is likely that archaeal viruses may account for a substantial fraction of the unclassified viral scaffolds in our study, especially in the archaea-predominating surface tailings.

Samples from both cores share a common depth-stratified pattern in the overall composition of geochemistry, prokaryotic communities, and viral communities (Fig. 3d-f). While it is unclear whether variations in viral communities were directly driven by their hosts or by geochemical changes along the tailings profiles, our results provided quantitative evidence that viral diversity increases with depth in the highly stratified mine tailings at this site (Fig. 1). Noteworthy, previous 16S rRNA gene surveys have identified pH as major driver of prokaryotic community composition at local or large scales in the extreme AMD and associated environments [37, 38]. Our current metagenomics analysis demonstrated that pH is also one of the major factors shaping the relatively under-studied viral world (Fig. 3b and c). That viral richness and normalized coverage increased with increasing pH along the depth profiles is somewhat expected because both viruses and their prokaryotic hosts tend to be sensitive to acidic pH [21, 22]. This would also explain the observed lower variability of both prokaryotic and viral populations at lower pH values (Fig. 3a and b).

Viruses depend on their prokaryotic hosts to successfully replicate. We hypothesized that viruses tend to be more temperate and symbiotic with hosts in extreme environmental conditions. This was supported by the significant negative correlations between the relative abundance of prophages and pH (Fig. 4). Our results are also consistent with previous studies which suggested that the lysogenic state should be favored under extreme conditions (for example, low nutrients, low productivity, or heat) [39]. This is a readily comprehensible pattern as lysogeny can enhance phage and host survival, particularly under adverse conditions [40]. Thus, the enrichment of prophages in the surface layers of the mine tailings not only might enable the detection of virus-host links, but also provides evidence for the potential preference of lysogenic viral lifestyle in more extreme environments.

Viral communities with diverse taxa in natural environments may exhibit distinct functional profiles in response to the varying biotic and abiotic factors [14, 41]. Comparative analysis of viral community gene profiles showed that metabolic patterns were significantly different between surface tailings and deeper layers (Fig. 5a) and, although found in all tailings layers, many indicator COGs had distinct, depth-related distribution (Additional file 1: Table S7). Archaeal viruses may be abundant in the surface tailings due to predominance of their potential hosts (archaea) in those layers (Fig. 2b). This speculation is supported by the finding that archaeal phage integrase (arCOG01244) were significantly abundant in the surface tailings (Additional file 1: Table S7). Thus, it is reasonable that viral indicator COGs in the surface tailings are more difficult to annotate due to the small number of isolated archaeal viruses [35, 36]. Compared with the more unidentifiable viral functions in the surface extreme environments, the category of functions in the deeper layers showed strong consistency with conventional metabolism and structure functions (Fig. 5b), suggesting that viruses in the less extreme deeper tailings layers are more similar to currently known viruses, which are isolated largely from non-extreme environments.

The role of viruses in regulating the sulfur cycle was recently described in deep ocean viral communities [4, 42]. Interestingly, our analyses showed that viral genes participating in the ASR process (COG0175) were significantly abundant in the deeper tailings layers (Fig. 5b and Fig. 6a), which were characterized by lower ratio of SO_4_^2−^/TS. In this process, sulfate is incorporated into adenosine-5′-phosphosulfate (APS), which is then activated by ATP to form PAPS that can be reduced to sulfite by PAPS reductase (COG0175), and further participated in the formation of many essential biomolecules like iron-sulfur (Fe–S) clusters, sulfur-containing amino acids, and cofactors [43]. Notably, previous study has reported ASR in members of *Acidithiobacillus*, an acidophilic *Gammaproteobacteria* genus often dominating AMD and associated environments [30]. The abundant viral ASR AMGs in the deeper layers possibly may facilitate their hosts to utilize sulfate in the oxygen-depleting environment, which in turn benefit the replication and reproduction of associated viruses. As AMD typically contains elevated levels of sulfate and metals due to oxidative dissolution of sulfide minerals, our findings of a potential contribution of viruses to the sulfate reduction process in the deeper part of the tailings impoundment have practical implications for AMD bioremediation.

## Conclusions

Although the field of viral ecology is rapidly evolving owing to recent developments of sequencing and bioinformatics methods, the viral communities populating various extreme environments remain relatively underexplored. Our comprehensive analysis of the mine tailings cores has revealed a largely novel, depth-stratified viral community that shows strong correlations with co-occurring prokaryotic assemblages and geochemical gradients. The environmental conditions associated with different oxidation stages of mine tailings (deep layers of the cores represent unaltered, pH-neutral tailings material whereas top layers represent highly oxidized and acidified tailings) apparently have a profound impact on the viral populations and their functions. Future simulated experiments of oxidative dissolution of sulfidic mine tailings or sulfide minerals, coupled with extensive time-series sampling and analysis, will provide more detailed insights into viral dynamics and their interplay with prokaryotic populations and geochemical conditions during the process of acid generation.

## Methods

### Study site, sampling, and physicochemical analyses

The Fankou Pb/Zn sulfidic mine tailings site (25° 2′ 56.5″ N, 113° 39′ 48.5″ E) is located in Shaoguan, Guangdong province, China. Extremely acidic, heavy metal-rich drainage is a persistent feature due to microbially mediated dissolution of sulfide minerals in the tailings at this site. Previous 16S rRNA gene surveys have documented vertical stratification of geochemistry and prokaryotic populations, with acidophilic archaea, mostly *Ferroplasma* spp. in the *Thermoplasmatales* predominant in the upper layers of tailings (oxidized zones and the oxidation front) [26]. Two tailings cores (inner diameter, 8 cm; length, 60 cm) were sampled from an area covered with AMD using a sampling collector in October 2017. After retrieval, the cores were immediately sectioned into distinct layers based on their physical feature and appearance (e.g., colors), yielding six layers for core A and five layers for core B (Additional file 2: Fig. S4). Each of the 11 tailings layers was collected in 50-ml sterile tubes, kept in an icebox and transported to the laboratory, where the samples were stored at 4 °C prior to subsequent analyses.

Air-dried subsamples were analyzed with standard methods for the determination of TOC (TOC-VCPH; Shimadzu, Columbia, MD) and TP (SmartChem; Westco Scientific Instruments Inc., Brookfield, CT). The pH and EC were measured in a 1:2.5 (w/v) aqueous solution using a pH meter and an EC meter. HCl-extractable ferrous iron was determined by the 1, 10-phenanthroline method at 530 nm [44], and sulfate (SO_4_^2−^) was measured by a BaSO_4_-based turbidimetric method [45]. Total concentrations of heavy metals (including Pb, Zn, Cu, Cr, Mn, and As) and TS were determined by inductively coupled plasma optical emission spectrometry (ICP-OES; Optima 2100DV, PerkinElmer, Wellesley, MA) and an elemental analyzer (Vario EL, Elementar, Germany), respectively.

### DNA extraction and 16S rRNA amplicon and metagenomic sequencing

Total community genomic DNA was extracted using the FastDNA Spin kit (MP Biomedicals, Irvine, CA) according to the manufacturer’s instructions. The V4 region of bacterial and archaeal 16S rRNA genes was amplified with prokaryotic universal primers F515 (5′-GTGCCAGCMGCCGCGGTAA-3′) and R806 (5′-GGACTACVSGGGTATCTAAT-3′) [46]. A sample-specific 8-bp error-correcting barcode was added to the reverse primer. PCR amplification was conducted in triplicate in 50-μl reaction mixtures following the thermal cycling procedure described previously [47, 48]. Replicate PCR reactions from each sample were pooled and concentrated and purified using a QIAquick Gel Extraction Kit (Qiagen, Chatsworth, CA). A single composite sample was prepared by combining an approximately equimolar amount of PCR product from each tailings sample and then sequenced on an Illumina MiSeq platform (Illumina, San Diego, CA) (250 bp, paired end reads). To obtain metagenomic data, extracted DNA was purified using a QIAquick Gel Extraction Kit (Qiagen, Chatsworth, CA), quantified with Qubit (Thermo Fisher Scientific, Australia). The total community DNA was used for library preparation with NEBNext Ultra II DNA Prep Kit (New England Biolabs, Ipswich, MA) and sequenced with MiSeq Reagent Kit v3 on an Illumina MiSeq platform (150 bp, paired end reads). Finally, 50-GB sequence data was obtained for each of the samples.

### Processing of 16S rRNA genes and metagenomic sequence data

Raw data of 16S rRNA genes were processed and analyzed with the Mothur software package (version 1.38.1) and QIIME (1.9.0) [49, 50]. Briefly, obtained short reads were noise reduced to minimize sequencing error by using the commands of “shhh.flows” and “pre.cluster” in Mothur [49]. Then, putative chimeric sequences were identified and removed by using Chimeric Uchime [51]. Pair-end reads were assembled via the “make.contigs” command, and the primers and barcodes in assembled sequences were removed using the “trim.seqs” commond [49]. Operational taxonomic units (OTUs) were identified by clustering assembled sequences at the 97% similarity level using UCLUST algorithm [51]. Taxonomic classification of the phylotypes was determined based on the Ribosomal Database Project at a default threshold of 80% [52]. Finally, the non-rarified OTU table (table of counts of OTUs on a per-sample basis with singleton OTUs excluded) and OTU taxonomy were converted to a “biom” format to obtain prokaryotic community composition at different taxonomic levels by using the script of “summarize_taxa_through_plots.py” in QIIME [50, 53, 54].

Metagenomic reads were quality filtered and trimmed using in-house Perl scripts “(https://github.com/eco-gaoshaom/in-house-scripts)”. A trim quality threshold of 20 was used and reads containing more than 5 “N” were discarded. All quality-controlled reads from a tailings core were cross-assembled using SPAdes 3.9.0 and kmers of 21, 33, 55, 77, 99, and 127 under the “--meta” mode [55]. Genes were predicted by Prodigal 2.6.3 (with the parameters set as “-p meta -g 11 -f gff -q -m -c”) [56], and functional annotation was performed through assignment of predicted proteins to the Pfam 32.0 [57], Kyoto Encyclopedia of Genes and Genomes (KEGG) database [58], and Non-supervised Orthologous Groups (eggNOG v5.0.0) [29]. Briefly, predicted proteins were compared to Pfam database by using the InterProscan 5.0 software with settings of “-appl Pfam -irplookup” and the lowest *E* value as the best hits. Additionally, blastp was used to assign viral proteins to KEGG and eggNOG database to get KO and COG terms (*E* value 10^−5^).

To access the dynamics of individual scaffolds and genes, sequencing reads from each library were mapped onto sequences using Bowtie2 with default parameters [59]. The normalized coverage for a given scaffold or gene was computed as the average scaffold or gene coverage (that is, the number of nucleotides mapped to the scaffold or gene divided by the scaffold or gene length) divided by the number of reads in a given library and multiplied by the mean value of the number of reads in the 11 libraries [5].

### Identification and clustering of viral scaffolds

Two methods were applied to identify viral scaffolds in the metagenomic assemblies: viral protein families generated with isolate reference viruses and viral scaffolds identified from a collection of geographically and ecologically diverse samples according to metadata from the Integrated Microbial Genomes with Microbiome (IMG/M) system [27], and VirSorter software based on the identification of viral hallmark genes, enrichment in hypothetical proteins, and other viral signatures [28]. First, viral protein family models were used as a bait to screen metagenomic scaffolds longer than 5 kb and then filtered by inspecting the number of genes covered with viral protein families, Pfams and KO terms, as previously described [27]. Next, metagenomic scaffolds longer than 3 kb were processed with VirSorter using the Viromes database [28]. The resulting scaffolds in the categories 1 and 2 were then manually curated as described previously [33]. For scaffolds in the categories 4 and 5, only predicted prophage regions were retained [9] and further manually curated to adjust the boundaries by removing annotated genes on scaffold edges beyond the first or last virus-specific gene (i.e., gene annotated with “capsid,” “phage,” “terminase,” “baseplate,” “base plate,” “prohead,” “virion,” “holing,” “virus,” “viral,” “tape measure,” “tapemeasure,” “neck,” “tail,” “p22,” “head,” “T4,” “prophage”) or integrase (eggNOG v5.0.0 database) [40]. Then, if the scaffolds predicted by viral protein families contain a prophage prediction, these scaffolds were removed from the predicted sequence pools identified by this method. Finally, to further avoid putative false positives, predicted scaffolds were considered viral if they satisfied one of the following: (1) contained virus-specific genes as defined above and (2) the total number of genes assigned as “unknown” (annotated with eggNOG v5.0.0 database) accounted for ≥ 80% of the total number of genes on the scaffold [27, 28].

All predicted viral scaffolds were clustered into viral OTUs (vOTUs) at approximately the species level using the parameters of 95% average nucleotide identity and 85% alignment fraction of the smallest scaffolds [32]. To place the viral scaffolds in the context of known viruses, a gene content-based network analysis was used to cluster viral scaffolds into viral clusters (VCs) at approximately the genus level [34]. Briefly, predicted proteins from viral scaffolds were clustered with predicted proteins from isolate reference viruses in the NCBI database (dsDNA viruses, ssDNA viruses, and retroviruses combined) [60] based on all versus-all blastp search with an *E* value of 10^−3^, and protein clusters were defined with the Markov clustering algorithm and processed using vConTACT v.2.0 [33, 61].

### Reconstruction of prokaryotic genomes and host prediction of viral scaffolds

All cross-assembled scaffolds longer than 2.5 kb were binned using MetaBAT v2.12.1 [62], MaxBin v2.2.2 [63], Abawaca v1.00 (https://github.com/CK7/abawaca), and Concoct v0.4.0 [64] with default parameters, considering tetranucleotide frequencies, scaffold coverage, and GC content, and then, the results were combined using DASTool [65]. Bins were further manually curated to obtain high-quality genomes using RefineM v0.0.24 [66]. In detail, the automatic binning methods may separate a “true” genome bin into two or more smaller, separate bins. Bins that shared a similar coverage range, GC content, and identical taxonomic classifications as determined by CheckM v1.0.7 [67] were grouped into a single bin. Additionally, scaffolds with incongruent taxonomic classification and incongruent 16S rRNA genes were removed as implemented in RefineM v0.0.24 [66]. The completeness and contamination of genome bins were assessed using CheckM v1.0.7 [67], and genomes estimated to be more than 50% complete and less than 10% contaminated were classified using the genome taxonomy database (GTDB-Tk v0.3.0) [68].

Viral scaffolds were putatively linked to their hosts in silico [69]. Briefly, these linkages were based on (1) shared genomic content between viral scaffolds and host genomes, (2) prophages identified in host genomes, and (3) sequence similarity between spacers in microbial CRISPR regions and in the viral scaffolds. All viral scaffolds were compared to the recovered host genomes (*E* value ≤ 10^−3^, bit score ≥ 50, alignment length ≥ 2.5 kb, and identity ≥ 70%) using blastn [4]. Viral sequences identified as prophage were matched to their corresponding host genomes. CRISPR spacers were recovered from metagenomic scaffolds using metaCRT with default parameters [70]. Extracted spacers were compared to viral scaffolds using blastn with thresholds of no mismatches over the whole spacer length and an *E* value ≤ 10^−10^ [1, 4].

### Analysis of AMGs

Viral genes predicted by Prodigal [56] were assigned to eggNOG v5.0.0 database [29] using blastp (threshold of 50 for bit score and 10^−5^ for *E* value). Viral AMGs assigned as COG0175 (PAPS reductase) were identified in the viral genomes [30] and then compared to the protein sequences in eggNOG v5.0.0 database [29] (blastp, threshold of 50 for bit score and 10^−3^ for *E* value) to recruit relevant reference sequences (up to 20 for each viral AMG sequence) [4]. These sets of viral AMGs and related protein sequences were then aligned with Muscle v3.8.31 [71] and filtered by TrimAL 1.2rev59 [72] to remove columns comprised of more than 95% gaps. Phylogenetic trees were reconstructed using RAxML (version 8.2.8 with the parameters set as “-f a -m GTRGAMMA -n boot -c 25 -p 12345 -x 12345”) [73]. The resulting newick file with the best tree topology determined as with the best likelihood score was uploaded to iTOL v4 for visualization and formatting [74].

### Statistical analyses

All statistical analyses were implemented with various packages within the statistical program R. Pearson correlations were performed using “rcorr” function (Hmisc package) to assess the relationships between the diversity of viruses, prokaryotes, and environmental variables in all samples. Bray–Curtis distances were used to construct the dissimilarity matrices for prokaryotic and viral community structure and function profiles, whereas Euclidean distances were calculated using standardized environmental variables (vegan 2.5-4). Permutational multivariate analysis of variance (“Adonis” function; 999 permutations) was used to test for significant differences between classified groups of samples (vegan 2.5-4). Mantel tests were performed to reveal the correlations between the dissimilarity matrices (vegan 2.5-4). Statistical significance of differences in normalized coverage of a given gene or COG between two datasets was determined using non-parametric Wilcoxon *t* test (unpaired), with confidence intervals at 99% significance and Benjamini–Hochberg correction (*P* < 0.05).

## Supplementary information

**Additional file 1: Table S1** Biotic and abiotic data for the tailings samples from the Fankou Pb/Zn Mine located in Guangdong Province, P.R. China. **Table S2** Detailed information of viral sequences identified in the tailings samples. **Table S3** Normalized coverage and taxonomic affiliation of the viral OTUs (vOTUs) identified in the tailings samples. **Table S4** 16S rRNA gene OTU table and taxonomy of the prokaryotes in the tailings samples. **Table S5** Virus-host linkages predicted by prophages and shared genomic matches with host genomes and by protospacer-spacer matches. **Table S6** Detailed functional and taxonomic descriptions of viral genes predicted with viral sequences identified in the tailings samples. Indicator COGs in the surface and deeper mine tailings are shown in red and blue, respectively. **Table S7** Normalized coverage and functional description of indicator COGs in the surface tailings (red) and deeper layers (blue). **Table S8** Detailed information of predicted viral sequences encoding indicator COGs in the surface tailings (red), deeper layers (blue), and both communities (black). **Table S9** Detailed functional description and sequence information of the seven viral genomes containing genes involved in assimilatory sulfate reduction (COG0175). **Table S10** Homologs of assimilatory sulfate reduction genes (COG0175) and corresponding taxonomy recruited from eggNOG v5.0.0 database.

**Additional file 2: Fig. S1** Pearson’s correlations between the biotic and abiotic factors with a color gradient denoting Pearson’s correlation coefficients and the number of asterisk corresponds to the Pearson’s statistic for the corresponding correlations (* 0.01 ≤ *P* < 0.05, ** 0.001 ≤ *P* < 0.01, and ***P < 0.001). **Fig. S2** Environmental drivers of prokaryotic and viral community composition. Pairwise comparisons of environmental factors are shown with a color gradient denoting Pearson’s correlation coefficients. Viral and prokaryotic taxonomic community composition was related to each environmental factor by Mantel tests. Edge width corresponds to the Mantel’s *r* statistic for the corresponding distance correlations, and edge color denotes the statistical significance. EC, electronic conductivity; TOC, total organic carbon; TP, total phosphorus; TS, total sulfur. **Fig. S3** Bar graphs showing the relative abundance of viruses encoding the indicator COGs in surface tailings (orange) and deeper layers (blue) and pie charts showing percent composition of viruses that encode the indicator COGs in each layers. **Fig. S4** Photos of the two tailings cores from the Fankou Pb/Zn Mine located in Guangdong Province.

## Data Availability

Raw reads of 16S rRNA gene amplicons and metagenomic sequencing are available for download from the Short Reads Archive with NCBI BioProject accession no. PRJNA515819.

## Abbreviations

EC: Electronic conductivity
TP: Total phosphorus
TS: Total sulfur
TOC: Total organic carbon
VC: Viral cluster
vOTU: Viral operational taxonomic unit
AMG: Auxiliary metabolic gene
COG: Clusters of orthologous group
NCBI: National Center for Biotechnology Information
eggNOG: Evolutionary genealogy of genes: Non-supervised Orthologous Groups
PCA: Principal component analysis
PCoA: Principle coordinate analysis
PCR: Polymerase chain reaction
ASR: Assimilatory sulfate reduction
APS: Adenosine-5′-phosphosulfate
PAPS: 3′-Phosphoadenosine 5′-phosphosulfate
