# Supplementary material for: Depth-related variability in viral communities in highly stratified sulfidic mine tailings
Source: Microbiome. 2020 Jun 9;8:89. doi: 10.1186/s40168-020-00848-3 (PMC7285708; doi:10.1186/s40168-020-00848-3)
Supplement: Supplementary file 3 — Additional file 2: Fig. S1 Pearson’s correlations between the biotic and abiotic factors with a color gradient denoting Pearson’s correlation coefficients and the number of asterisk corresponds to the Pearson’s statistic for the corresponding correlations (* 0.01 ≤ P < 0.05, ** 0.001 ≤ P < 0.01, and ***P < 0.001). Fig. S2 Environmental drivers of prokaryotic and viral community composition. Pairwise comparisons of environmental factors are shown with a color gradient denoting Pearson’s correlation coefficients. Viral and prokaryotic taxonomic community composition was related to each environmental factor by Mantel tests. Edge width corresponds to the Mantel’s r statistic for the corresponding distance correlations, and edge color denotes the statistical significance. EC, electronic conductivity; TOC, total organic carbon; TP, total phosphorus; TS, total sulfur. Fig. S3 Bar graphs showing the relative abundance of viruses encoding the indicator COGs in surface tailings (orange) and deeper layers (blue) and pie charts showing percent composition of viruses that encode the indicator COGs in each layers. Fig. S4 Photos of the two tailings cores from the Fankou Pb/Zn Mine located in Guangdong Province. [file 40168_2020_848_MOESM2_ESM.docx]

**
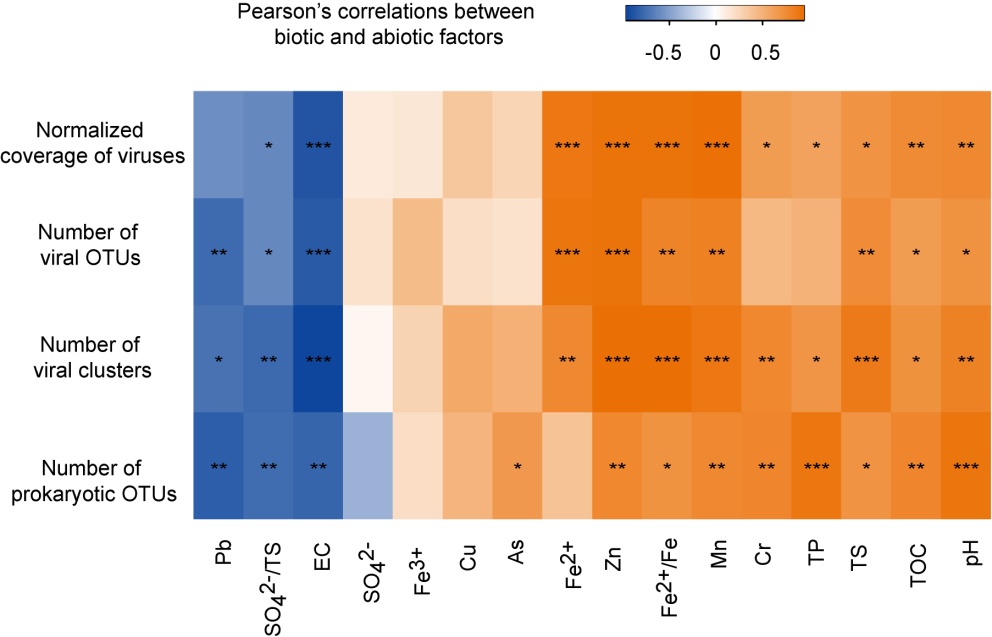
**

**Fig. S1** Pearson’s correlations between the biotic and abiotic factors with a color gradient denoting Pearson’s correlation coefficients and the number of asterisk corresponds to the Pearson’s statistic for the corresponding correlations (*0.01 ≤ *P* < 0.05, **0.001 ≤ *P* < 0.01, and ****P* < 0.001).

**
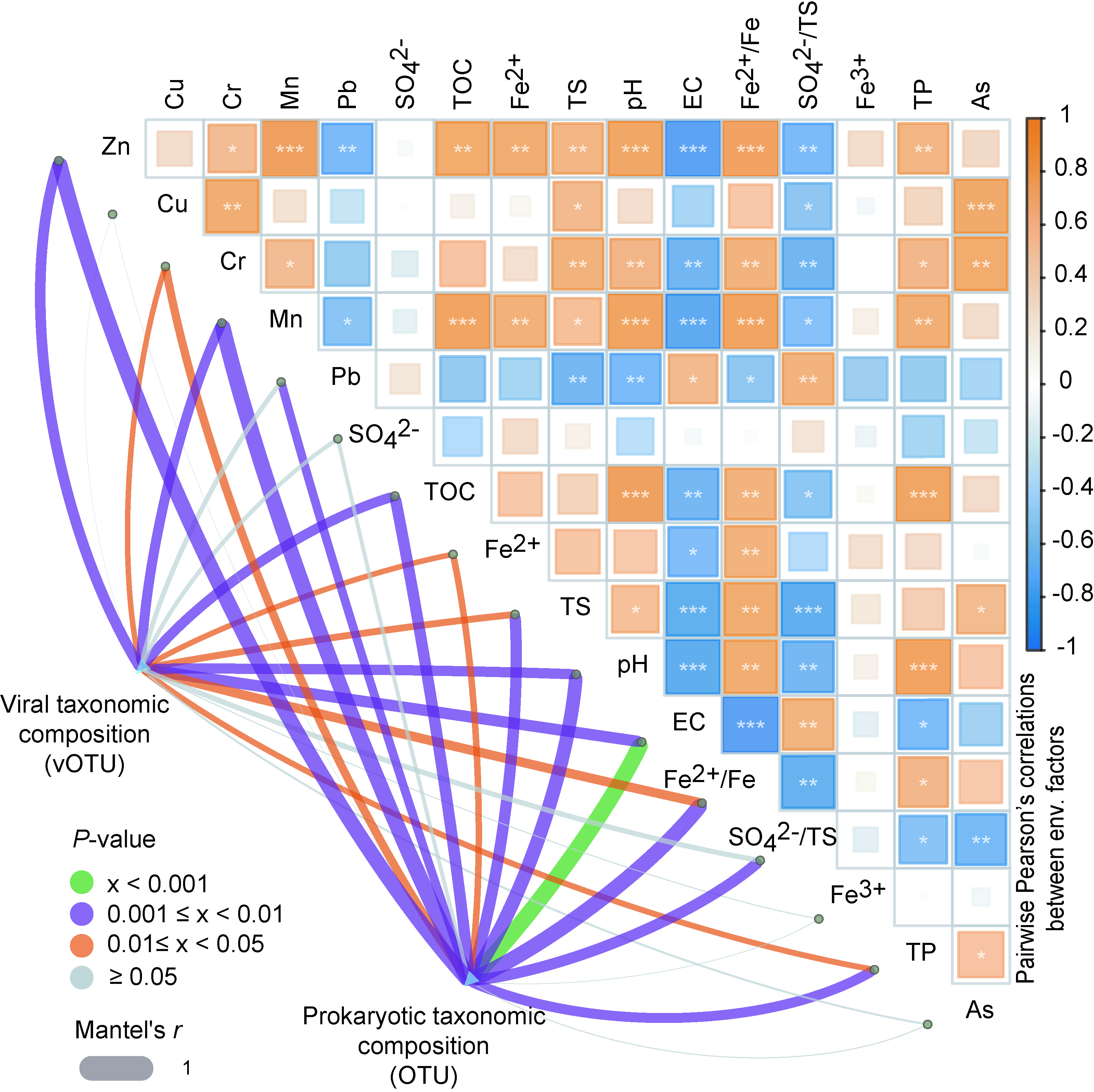
**

**Fig. S2** Environmental drivers of prokaryotic and viral community composition. Pairwise comparisons of environmental factors are shown with a color gradient denoting Pearson’s correlation coefficients. Viral and prokaryotic taxonomic community composition was related to each environmental factor by Mantel tests. Edge width corresponds to the Mantel’s *r* statistic for the corresponding distance correlations, and edge color denotes the statistical significance. EC, electronic conductivity; TOC, total organic carbon; TP, total phosphorus; TS, total sulfur.


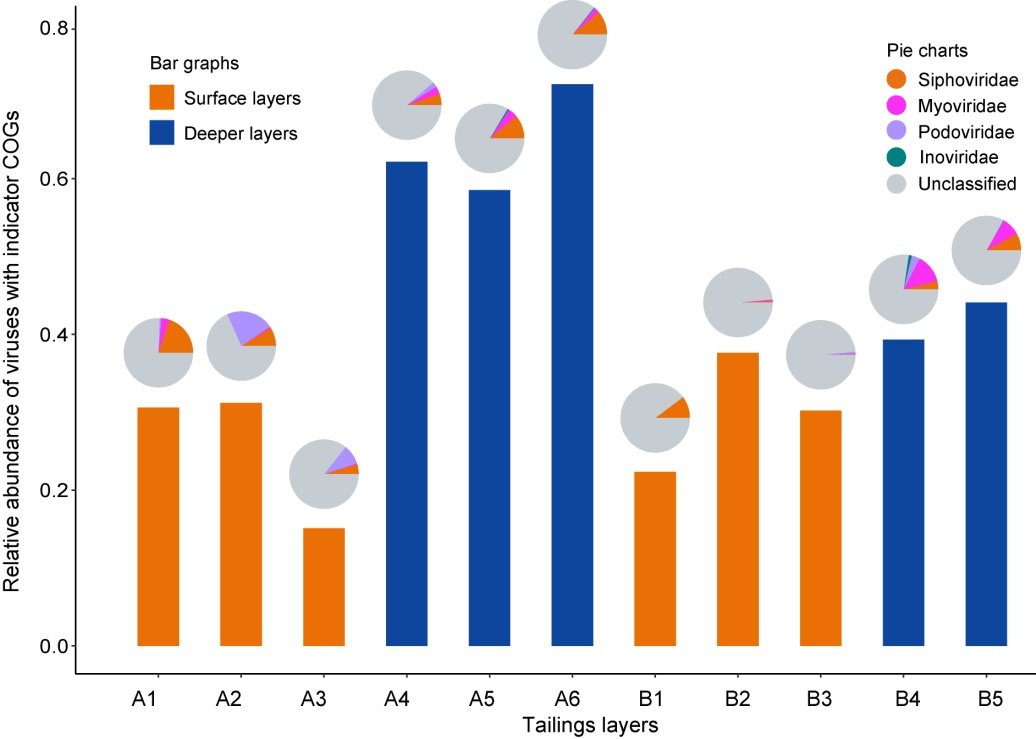


**Fig. S3** Bar graphs showing the relative abundance of viruses encoding the indicator COGs in surface tailings (orange) and deeper layers (blue) and pie charts showing percent composition of viruses that encode the indicator COGs in each layers.

**
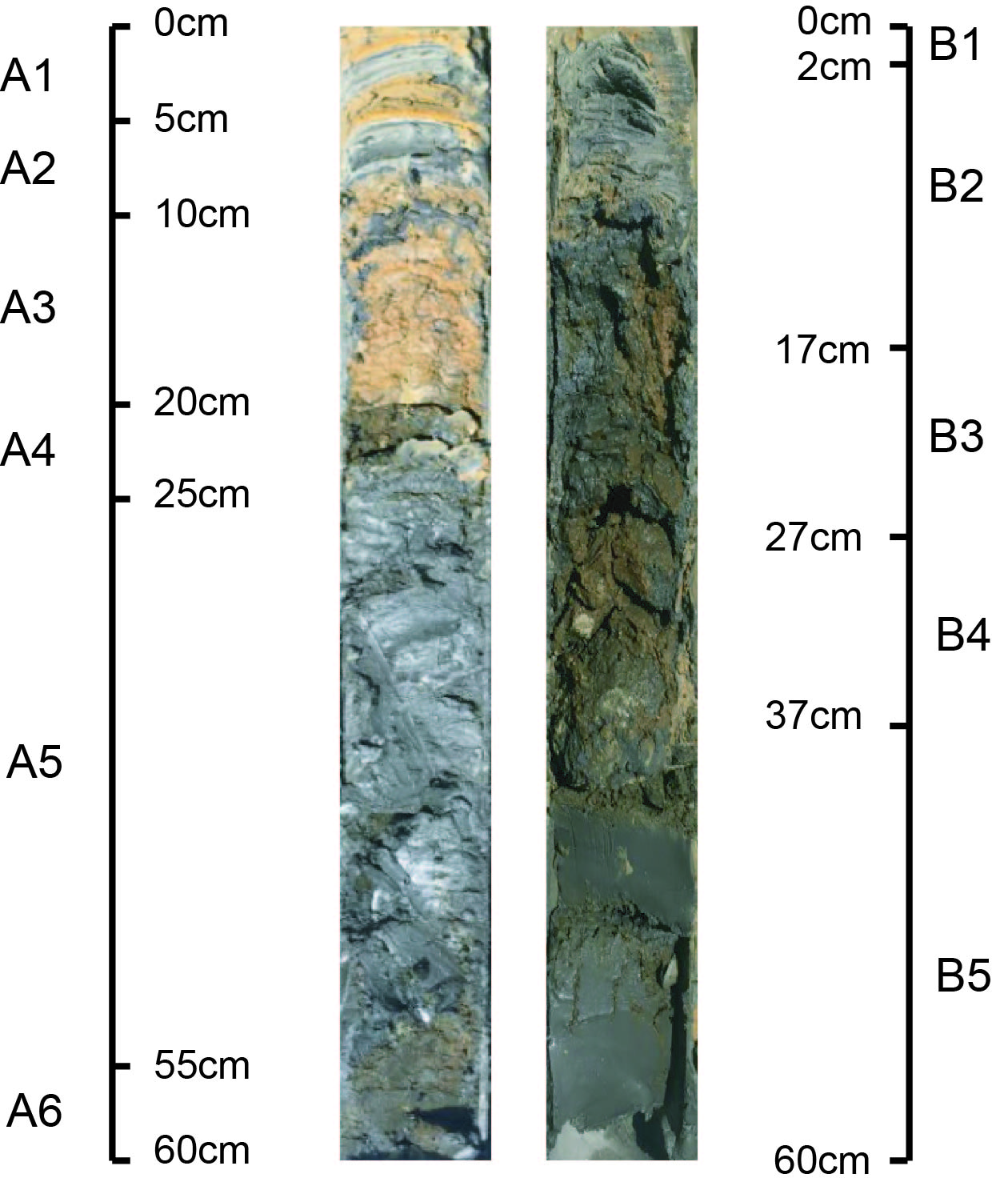
Fig. S4** Photos of the two tailings cores from the Fankou Pb/Zn Mine located in Guangdong Province.
